# Supplementary material for: Learning to stand with sensorimotor delays generalizes across directions and from hand to leg effectors
Source: Commun Biol. 2024 Mar 29;7:384. doi: 10.1038/s42003-024-06029-4 (PMC10980713; doi:10.1038/s42003-024-06029-4)
Supplement: Supplementary file 2 — Supplementary Information [file 42003_2024_6029_MOESM2_ESM.pdf]

Supplementary Information for

Learning to stand with sensorimotor delays generalizes across directions and from hand to leg effectors

Brandon G. Rasman, Jean-Sébastien Blouin, Amin M. Nasrabadi, Remco van Woerkom, Maarten A Frens, Patrick A. Forbes*

*Corresponding author: Patrick A. Forbes, p.forbes@erasmusmc.nl, Department of Neuroscience, Erasmus MC, University Medical Center Rotterdam, Rotterdam, The Netherlands

This PDF file includes:

Supplementary Notes 1-4
Supplementary Figures 1-4
Supplementary Table 1

Supporting Information

Supplementary Note 1: Experiment 1: Relative transfer estimated from pre- to post-learning delay trials

The main analysis of the pre- and post-learning delay trials in Experiment 1 (see Fig. 2 and Fig. 3 in main manuscript) demonstrated that after training in one direction, participants improved their balance performance when balancing with an imposed delay in both the trained and untrained directions. To further examine how training to balance with a delay in a single direction transferred to the orthogonal (untrained) direction, we calculated the relative improvement (i.e., percent improvement) between pre- and post-learning delay trials for both the trained and untrained conditions on a participant-by-participant basis. Relative improvement was calculated for angular velocity variance, percent time within the limits and ankle torque SD metrics. Here, we compared the relative improvement (i.e., percent improvement) of the trained and untrained conditions across training groups for each delay condition (i.e., AP and ML delay trials) by using two-tailed paired t-tests (Bonferroni corrected).

When assessing the percent improvements in the AP-delay trials, the AP training group demonstrated greater relative improvement than the ML training group (Supplementary Fig. 1). There was a significant difference between AP and ML groups for all metrics: AP angular velocity variance improvement (AP training group: 77 ± 4 % improvement vs ML training group: 35 ± 13 %, $p < 0.01$), AP percent time within limits (AP training group: 53 ± 3 % vs ML training group: 17 ± 2 %, $p < 0.001$) and AP ankle torque SD (AP training group: 53 ± 5 % vs ML training group: 23 ± 7 %, $p < 0.05$). Similarly, when assessing the percent improvement in the ML-delay trials, the ML training group demonstrated greater relative improvement than the AP training group. There was a significant difference between ML and AP groups for all metrics: ML angular velocity variance (ML training group: 79 ± 4 % vs AP training group: 35 ± 8 %, $p < 0.001$), ML percent time within limits (ML training group: 42 ± 4 % vs 21 ± 4 %, $p < 0.01$), and ML ankle torque SD (ML training group: 53 ± 5 % vs AP training group: 20 ± 8 %, $p < 0.05$). These differences in relative improvements demonstrate that balance learning was largest in the condition that was trained.

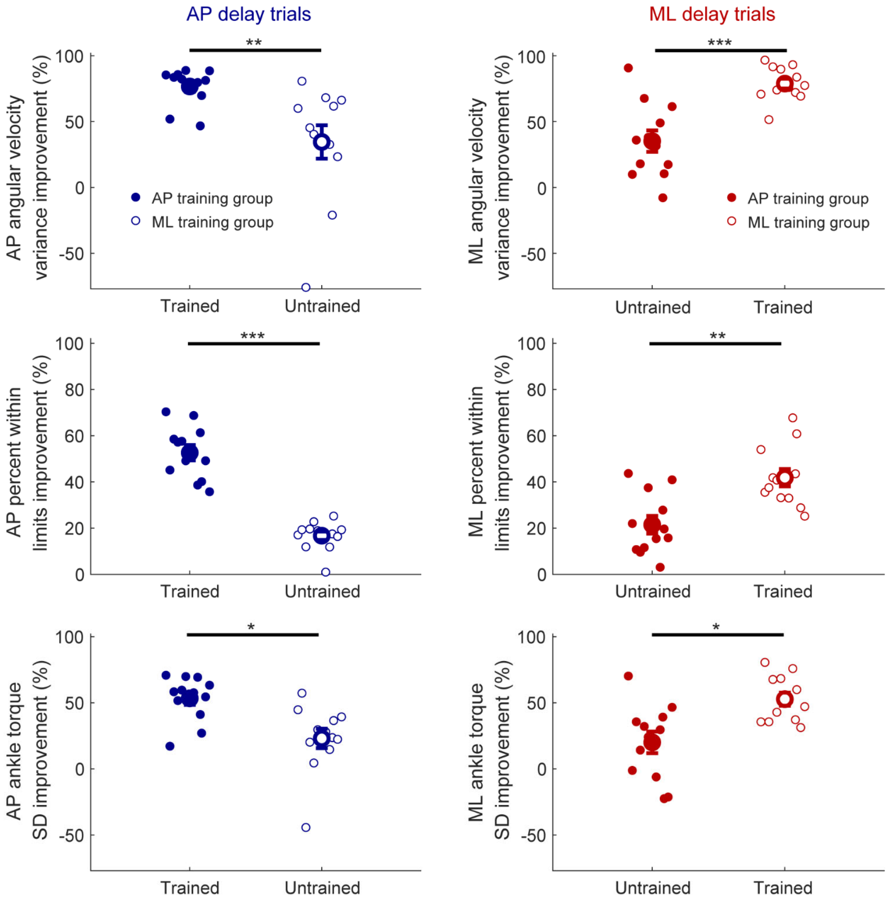

Supplementary Fig. 1: Relative improvements between pre- and post-learning delay trials in Experiment 1. Percent improvement between pre- and post-learning trials are presented for angular velocity variance, percent time within limits, and ankle torque SD for both AP-delay trials (data in blue) and ML-delay trials (data in red). Small circles are individual participants and large circles are group averages (*n* = 12 for each group) with SEM error bars. Closed circles represent the AP training group and open circles represent the ML training group. Regardless of which direction was trained (AP or ML), balance improvements were observed for both AP-delay and ML-delay conditions (see Main Manuscript). However, there were significant differences between trained and untrained conditions, such that relative improvements were greater in the trained condition. * indicates $p < 0.05$, ** indicates $p < 0.01$, *** indicates $p < 0.001$).

Supplementary Note 2: Experiment 1: Changes in the frequency distribution of angular velocity and torque during bi-directional baseline standing

When comparing the pre- and post-learning baseline trials when balancing in both directions (i.e., AP-baseline/ML-baseline), we observed specific changes in the frequency distribution of power in both angular velocity and torque signals. A peak in autospectra power emerged at ~1.4 Hz while the power at frequencies from 0.4-1.2 Hz slightly decreased. Notably, this change was only observed in the direction that was trained (i.e., AP direction for AP training group and ML direction for the ML training group), even when participants were free to balance in both directions (see Supplementary Fig. 2). This aligned with the results observed in single direction baseline

conditions (see Fig. 4 and Fig. 5 in main manuscript) and suggests that the aftereffect of learning is limited to the direction of balance that was trained.

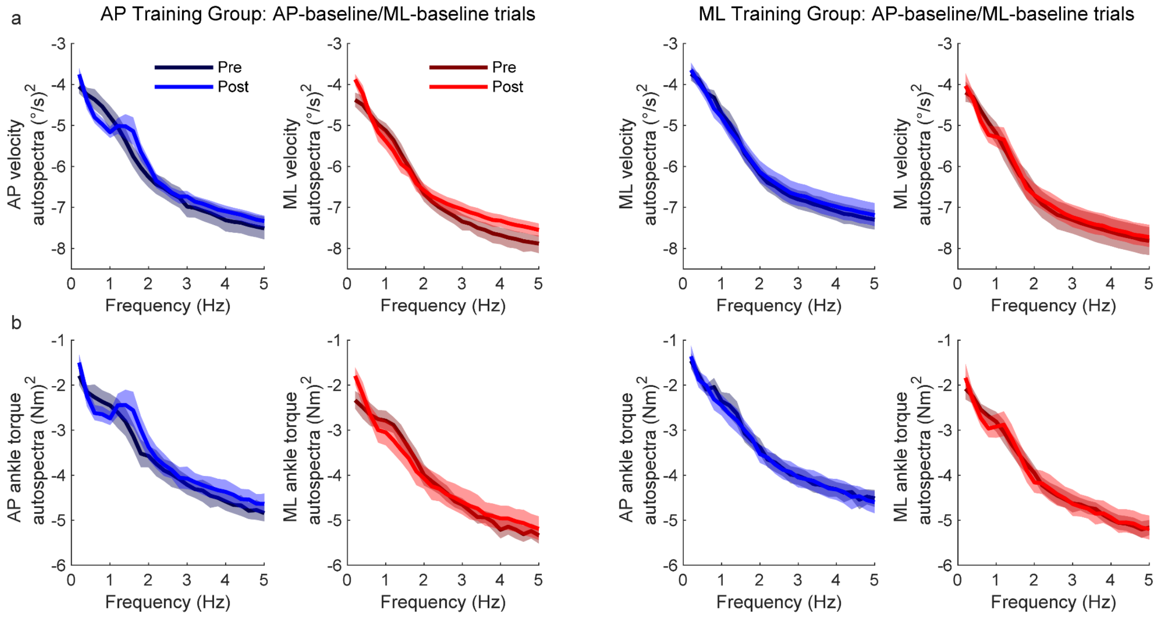

Supplementary Fig. 2: Frequency characteristics of angular velocity and ankle torque signals in AP-baseline/ML-baseline pre- and post-learning trials (Experiment 1). a Autospectra of angular velocity signals extracted from pre- and post-learning baseline trials. **b** Autospectra of ankle torque signals extracted from pre- and post-learning baseline trials. For all panels, data in blue represent AP-baseline trials whereas data in red represent ML-baseline trials. Darker lines represent the mean of pre-learning trials, while lighter lines represent the mean of post-learning trials. Shaded regions around the means represent the bootstrapped 95% confidence interval.

Supplementary Note 3: Experiment 1: No change in whole-body behavior with imposed delays after control group training

Ten participants performed a control experiment that was nearly identical to the Experiment 1 training experiment (pre- and post-learning delay trials with a 350 ms delay), with the training session being replaced by 60 minutes of baseline standing on the robot in the AP direction. This was to test for whether exposure to balancing on the robot (and not training with delays) was responsible for differences between pre- *vs* post-learning observed in participants from Experiment 1 who trained with imposed delays (presented in main manuscript). When comparing the pre- vs. post-learning trials with an imposed delay for this control group, there were no differences in balance behavior (angular velocity variance and percent within limits presented in Supplementary Fig. 3). For AP-delay trials, there was no change in AP angular velocity variance (pre: 18.4 ± 2.7 (°/s)^2 vs post: 18.1 ± 2.7 (°/s)^2; p = 0.87), no change in percent time within AP limits (pre: 67 ± 2% vs post: 68 ± 2%; p = 0.10), and no change in AP ankle torque SD (pre: 41.6 ± 3.1 (Nm) vs post: 42.5 ± 3.3 (Nm); p = 0.63). Similarly, for ML-delay trials, there was no change in ML angular velocity variance (pre: 13.5 ± 2.2 (°/s)^2 vs post: 14.0 ± 1.5 (°/s)^2, p = 0.93), no change in percent time within ML limits (65% ± 1% to 66% ± 2%; p = 0.68), and no change in ML ankle torque SD (pre: 36.3 ± 4.6 (Nm) vs post: 36.6 ± 3.4 (Nm); p = 0.93).

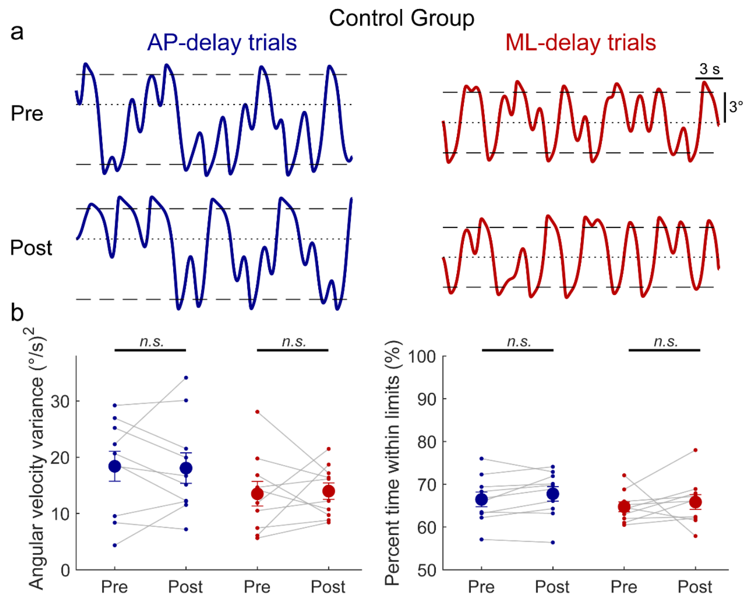

Supplementary Fig. 3: Standing balance behavior with an imposed 350 ms delay for participants from the control group (Experiment 1). a Whole-body angular position (°) traces from a representative participant balancing in the AP-delay and ML-delay trials. The top and bottom rows illustrate pre- and post-learning delay trials, respectively. The experimental design was nearly identical to the training groups in Experiment 1, with the control group performing a 60-minute session standing in the AP-baseline/ML-fixed condition instead of training with an imposed delay. Dashed lines represent the virtual position limits for AP (6° anterior, 3° posterior) and ML (3° left, 3° right) directions. Dotted lines represent the 0° position for all conditions. **b** Angular velocity variance and percent time within the limits in the pre- and post-learning AP-delay and ML-delay trials. Small circles connected with thin lines are individual participants and larger filled circles are group averages (*n* = 10) with accompanying SEM error bars. No changes were observed between pre- and post-learning trials in angular velocity variance or percent time within limits measures. n.s. indicates not significant. For all panels, data in blue represent AP-delay trials whereas data in red represent ML-delay trials.

Supplementary Note 4: Experiment 2: Evidence of biomechanical interactions in the control of balance influencing standing behavior

The aim of Experiment 2 was to determine how existing biomechanical interactions between AP and ML directions of standing balance influence postural behavior, particularly in the context of balancing with imposed delays. Our primary comparison of movement behavior in the direction orthogonal to an imposed delay (see Fig. 6 in main manuscript) revealed that standing movement variability increases in both directions of balance when a delay was imposed in a single direction. To further examine how biomechanical interactions influenced multidirectional standing behavior, we also compared angular velocity variance between several conditions in Experiment 2 where participants balanced freely in only one or both directions (Supplementary Fig. 4 and all statistical comparisons are presented in Supplementary Table 1). When balancing under baseline conditions (i.e., ~4 ms delay), movement behavior was similar whether

participants were balancing freely in one or both directions of standing (i.e., AP baseline/ML fixed vs AP baseline/ML baseline). There were also no differences in the disruptive effect of a delay in the direction it was imposed with 1) balance restricted in the orthogonal direction (i.e., AP-delay/ML fixed vs AP-delay/ML-baseline) or 2) with a delay also imposed in the orthogonal direction (i.e., AP-delay/ML-baseline vs AP-delay/ML-delay). Overall, these comparisons demonstrate that while there is evidence of biomechanical interactions influencing multidirectional standing behavior, they were only observed in the direction of balance that is orthogonal (and not delayed) to the direction of the imposed delay.

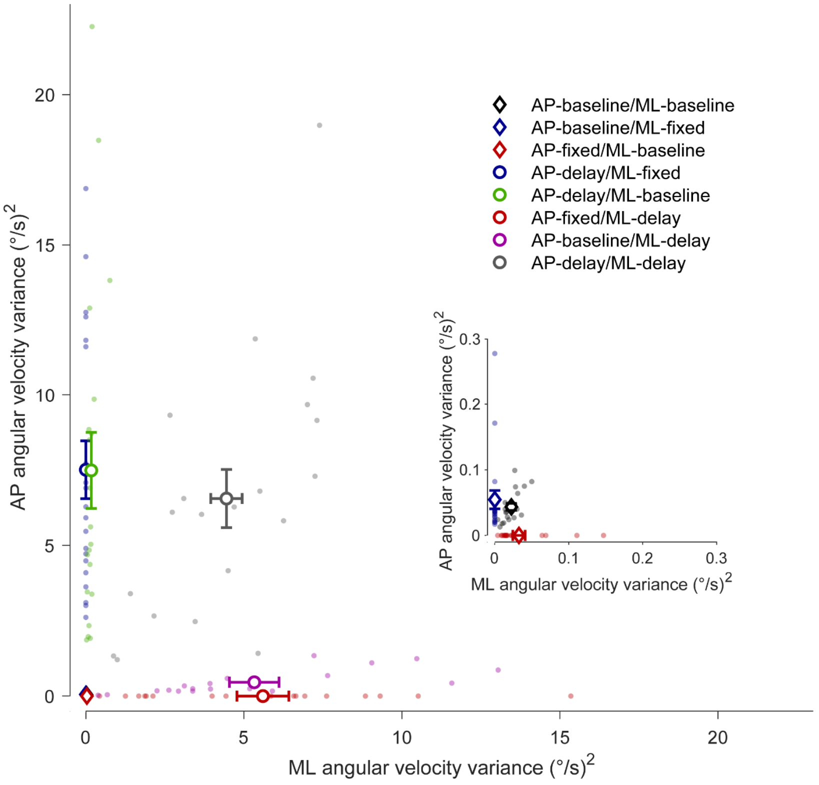

Supplementary Fig. 4: Standing balance behavior across imposed delay and control conditions (i.e., fixed/free) in different directions during Experiment 2. Single participant and group (n = 20) averages of angular velocity variance during the eight balance conditions. Open circles represent imposed delay (200 ms) trials. Diamonds representing the baseline conditions appear overlapping due to differences in scale and are therefore also presented as an inset. When a balance direction was fixed, angular velocity variance in that direction was zero. Small circles represent individual participants. Large circles and error bars represent group means and SEMs, respectively. When balancing under baseline conditions (i.e., 4 ms delay), movement behavior was similar between balancing freely in a single or both directions (i.e., black diamond *vs* blue diamond and black diamond *vs* red diamond). When balancing freely in both directions, an imposed delay in one direction increased movement variability in both the delayed and orthogonal direction (green circle *vs* black diamond and purple circle *vs* black diamond; also see Fig. 6 in main manuscript). There were no differences in the

disruptive effect of a delay in the direction it was imposed when balance was restricted in a single direction (blue circle *vs* green circle and red circle *vs* purple circle) or when delays were imposed in both directions (green circle vs grey circle and purple circle vs grey circle). Results of all statistical comparisons are presented in Supplementary Table 1.

Supplementary Table 1: Statistical results from Experiment 2

Pairwise comparisons	t statistic	p value
AP angular velocity variance (°/s)^2		
AP-baseline/ML-delay *vs.* AP-baseline/ML-baseline	4.78	< 0.001
AP-baseline/ML-fixed *vs.* AP-baseline/ML-baseline	0.79	= 0.44
AP-delay/ML-fixed *vs.* AP-delay/ML-baseline	0.03	= 0.98
AP-delay/ML-baseline *vs.* AP-delay/ML-delay	0.74	= 0.47
ML angular velocity variance (°/s)^2		
AP-delay/ML-baseline *vs.* AP-baseline/ML-baseline	4.10	< 0.001
AP-fixed/ML-baseline *vs.* AP-baseline/ML-baseline	1.31	= 0.21
AP-fixed/ML-delay *vs.* AP-baseline/ML-delay	0.50	= 0.62
AP-baseline/ML-delay *vs.* AP-delay/ML-delay	1.92	= 0.07
All statistical comparisons were performed using two-tailed paired t-tests with Bonferonni corrections for multiple comparisons (threshold p = 0.0125). A 200 ms delay was used for the balance trials with imposed delays.		
