# Supplementary material for: Learning to stand with sensorimotor delays generalizes across directions and from hand to leg effectors
Source: Commun Biol. 2024 Mar 29;7:384. doi: 10.1038/s42003-024-06029-4 (PMC10980713; doi:10.1038/s42003-024-06029-4)
Supplement: Supplementary file 3 — Reporting Summary [file 42003_2024_6029_MOESM3_ESM.pdf]

nature portfolio

Corresponding author(s): Dr. Patrick A Forbes

Last updated by author(s): Feb 18, 2024

Reporting Summary

Nature Portfolio wishes to improve the reproducibility of the work that we publish. This form provides structure for consistency and transparency in reporting. For further information on Nature Portfolio policies, see our Editorial Policies and the Editorial Policy Checklist.

Statistics

For all statistical analyses, confirm that the following items are present in the figure legend, table legend, main text, or Methods section.

n/a	Confirmed	
☐	☒	The exact sample size (*n*) for each experimental group/condition, given as a discrete number and unit of measurement
☐	☒	A statement on whether measurements were taken from distinct samples or whether the same sample was measured repeatedly
☐	☒	The statistical test(s) used AND whether they are one- or two-sided<br>*Only common tests should be described solely by name; describe more complex techniques in the Methods section.*
☐	☒	A description of all covariates tested
☐	☒	A description of any assumptions or corrections, such as tests of normality and adjustment for multiple comparisons
☐	☒	A full description of the statistical parameters including central tendency (e.g. means) or other basic estimates (e.g. regression coefficient) AND variation (e.g. standard deviation) or associated estimates of uncertainty (e.g. confidence intervals)
☐	☒	For null hypothesis testing, the test statistic (e.g. *F*, *t*, *r*) with confidence intervals, effect sizes, degrees of freedom and *P* value noted<br>*Give P values as exact values whenever suitable.*
☒	☐	For Bayesian analysis, information on the choice of priors and Markov chain Monte Carlo settings
☒	☐	For hierarchical and complex designs, identification of the appropriate level for tests and full reporting of outcomes
☒	☐	Estimates of effect sizes (e.g. Cohen's *d*, Pearson's *r*), indicating how they were calculated

Our web collection on statistics for biologists contains articles on many of the points above.

Software and code

Policy information about availability of computer code

Data collection	Custom designed LabVIEW 2019 code was used to operate the robotic balance simulator and collect data in the current study.
Data analysis	All data analyses were conducted using MATLAB R2022a.

For manuscripts utilizing custom algorithms or software that are central to the research but not yet described in published literature, software must be made available to editors and reviewers. We strongly encourage code deposition in a community repository (e.g. GitHub). See the Nature Portfolio guidelines for submitting code & software for further information.

Data

Policy information about availability of data

All manuscripts must include a data availability statement. This statement should provide the following information, where applicable:

- Accession codes, unique identifiers, or web links for publicly available datasets
- A description of any restrictions on data availability
- For clinical datasets or third party data, please ensure that the statement adheres to our policy

The source Matlab data and code files that support the findings of this study and generate the main figure results are available in "Data and code for "Learning to stand with sensorimotor delays generalizes across directions and from hand to leg effectors"" https://doi.org/10.34894/AT8YSZ, DataverseNL. Datasets generated and analyzed during the study are available from the corresponding author upon reasonable request.

Human research participants

Policy information about studies involving human research participants and Sex and Gender in Research.

Reporting on sex and gender	Neither sex nor gender was considered in the study design. Sex was self-reported. A total of 66 volunteers participated in this study (30 females, 36 males).
Population characteristics	The research sample was composed mainly of undergraduate and graduate students from Erasmus MC. The mean age of participants was 25.5 (SD: 3.9) years old.
Recruitment	Participants were recruited by an announcement with the invitation to participate in the experiment which was placed around the Erasmus MC facility.
Ethics oversight	This study was approved by the Medical Research Ethics Committee Erasmus MC (NL76700.078.21)

Note that full information on the approval of the study protocol must also be provided in the manuscript.

Field-specific reporting

Please select the one below that is the best fit for your research. If you are not sure, read the appropriate sections before making your selection.

☒ Life sciences ☐ Behavioural & social sciences ☐ Ecological, evolutionary & environmental sciences

For a reference copy of the document with all sections, see nature.com/documents/nr-reporting-summary-flat.pdf

Life sciences study design

All studies must disclose on these points even when the disclosure is negative.

Sample size	No sample-size calculation was performed. The sample size was chosen based on previous experiments performed in the lab and in line with other studies evaluating human balance control in different environmental conditions. See for example:<br>Day BL, Steiger MJ, Thompson PD, Marsden CD. Effect of vision and stance width on human body motion when standing: implications for afferent control of lateral sway. J Physiol 469, 479-499 (1993).<br>Kiemel T, Zhang Y, Jeka JJ. Identification of neural feedback for upright stance in humans: stabilization rather than sway minimization. J Neurosci 31, 15144-15153 (2011).<br>Choi JT, Bastian AJ. Adaptation reveals independent control networks for human walking. Nat Neurosci 10, (2007).<br>Rasman BG, et al. Learning to stand with unexpected sensorimotor delays. Elife 10, (2021).
Data exclusions	There were no data exclusions.
Replication	Several analyzes were performed to ensure the validity of the presented results. Different cohorts of participants were analyzed validating the obtained outcomes.
Randomization	Experiments were performed in chronological order. Therefore, participants were allocated to a particular experiment based on the time of recruitment (i.e., the experiment which was being conducted at the time of recruitment). In Experiment 1, participants were randomly allocated to either the AP or ML training group.
Blinding	Participants were assigned randomly to the groups but the investigators were not blind to this allocation. Blinding was not necessary for this study as the investigators awareness of group allocation could not influence the results.

Reporting for specific materials, systems and methods

We require information from authors about some types of materials, experimental systems and methods used in many studies. Here, indicate whether each material, system or method listed is relevant to your study. If you are not sure if a list item applies to your research, read the appropriate section before selecting a response.

Materials & experimental systems

n/a	Involved in the study	
☒	☐	Antibodies
☒	☐	Eukaryotic cell lines
☒	☐	Palaeontology and archaeology
☒	☐	Animals and other organisms
☒	☐	Clinical data
☒	☐	Dual use research of concern

Methods

n/a	Involved in the study	
☒	☐	ChIP-seq
☒	☐	Flow cytometry
☒	☐	MRI-based neuroimaging
